# Skeletal Metastasis of Unknown Primary Origin at the Initial Visit: A Retrospective Analysis of 286 Cases

**DOI:** 10.1371/journal.pone.0129428

**Published:** 2015-06-26

**Authors:** Tatsuya Takagi, Hirohisa Katagiri, Yongji Kim, Yoshiyuki Suehara, Daisuke Kubota, Keisuke Akaike, Midori Ishii, Kenta Mukaihara, Taketo Okubo, Hideki Murata, Mitsuru Takahashi, Kazuo Kaneko, Tsuyoshi Saito

**Affiliations:** 1 Department of Orthopaedic Surgery, Juntendo University School of Medicine, Tokyo, Japan; 2 Department of Orthopaedic Surgery, Shizuoka Cancer Center, Shizuoka, Japan; 3 Department of Human Pathology, Juntendo University School of Medicine, Tokyo, Japan; University of North Carolina School of Medicine, UNITED STATES

## Abstract

**Background:**

Skeletal metastasis is a common metastatic event for several carcinomas, and the treatment for skeletal metastasis of unknown primary (SMUP) are a critical issue in cancer therapy. Making a diagnosis of the primary site is the most crucial step in the treatment of SMUP; however, the procedures are sometimes difficult and time-consuming, and the primary site often remains unknown. Therefore, to establish optimal diagnostic strategies and elucidate the overall survival rates of SMUP, we conducted this retrospective study.

**Methods:**

We retrospectively analyzed the clinical data for 286 SMUP cases from a total of 2,641 patients with skeletal metastases who were treated between 2002 and 2014 at our initiations.

**Results:**

The primary sites were identified in 254/286 patients (88.8%), while 32 (11.2%) primary sites were not detected by our diagnostic strategies. Lung cancer was identified in 72 (25.2%) cases, and was the most frequently observed primary lesion. The median survival time of the SMUP patients was 20.0 months, while the median survival times of solitary bone metastasis cases and multi-bone metastasis cases were 39.0 months and 16.0 months, respectively. The median survival times of prostate cancer cases was over 120 months, that of patients with primary lung cancers was 9.0 months and the median survival time of cases who were finally diagnosed with an unknown primary was 11.0 months.

**Conclusions:**

We believe that our study would contribute to establishing an optimal strategy for diagnosing the primary site in SMUP patients, and our data provide definite indications for the survival times for different SMUP situations.

## Introduction

Skeletal metastasis is a common situation encountered in the treatment of various cancers and myelogenous disorders. At the initial visit, 7.8–21.7% of patients who have developed bone metastases have an unknown primary tumor [1, 2]. In cases of skeletal metastasis of unknown primary (SMUP), the diagnosis of the cancer of origin is difficult, and clinicians often take a long time to make a diagnosis of the primary site of the bone metastasis. Furthermore, there are some cases in which the primary site of the skeletal lesions cannot be identified even after extensive examinations. The delayed diagnosis has negative effects on the prognosis and also enhances the risk of skeletal-related events (SRE), including fractures or spinal cord compression. Therefore, it is critical to quickly identify the primary site from which the skeletal metastasis originated using optimal diagnostic strategies. It is also helpful to know the survival times expected for each type of SMUP patient, because this may alter the treatment strategy.

However, most of the existing reports on metastatic cancer of unknown primary site have not focused on these issues. Furthermore, the few reports which have focused on the SMUP employed small cohorts [1–3] and did not describe the survival of these SMUP cohorts, which include many different primary sites. Therefore, we conducted a retrospective study of 286 patients with SMUP to clarify the epidemiology of the primary sites, the overall survival rates and useful diagnostic tools enabling the prompt detection of the primary cancer.

## Materials and Methods

### 2.1 Patients

We retrospectively analyzed the clinical data of 286 SMUP patients out of a total of 2,641 skeletal metastases patients who were treated between 2002 and 2014 at Shizuoka Cancer Center and Juntendo University Hospital. We excluded patients who had past histories of cancer or tumors within the past 15 years or who were under 15 years old. The patients with SMUP were retrospectively analyzed with the clinical data. This study included 174 males and 112 females with an average age of 65.2 years old (range, 25–93 years old). All of the cases had osteolytic and/or sclerotic skeletal lesions on the plain radiographs.

The study was approved by the ethics committees at our institutions.

### 2.2 Examinations and clinical information

Our diagnostic strategy consisted of nine clinical steps including: Step 1) medical history-taking, Step 2) physical examinations, Step 3) chest X-rays, Step 4) blood tests (including tumor markers (CEA (carcinoembryonic antigen), CA19-9 (carbohydrate antigen 19–9), AFP (alpha-fetoprotein), PSA (prostate-specific antigen), PIVKA-II (protein induced by vitamin K absence or antagonist-II), IL-2 (interleukin-2) and IEP (immune electrophoresis)), Step 5) whole computed tomography (CT) scans, Step 6) bone biopsies, Step 7) origin examinations consisting of specific imaging studies (including thyroid echo, mammography, gastric fiberscopy and colon fiberscopy) and origin biopsies, Step 8) bone marrow punctures (BMP), Step 9) positron emission tomography (PET) scans. We also divided these steps into two categories; steps 1~5 as “common examinations”, and steps 6~9 as “advanced examinations”. Bone scans were also performed to detect the precise location of the metastases. We divided the patient status using the performance status (PS 0–4) of the ECOG (Eastern Cooperative Oncology Group), and obtained the additional clinical information from the medical records.

### 2.3 Statistical analysis

The overall survival times were calculated from the first treatment until death from tumor-specific causes. All time-to-event endpoints were computed by the Kaplan-Meier method. Potential prognostic factors were identified by a univariate analysis using the log-rank test. The relationships between the PS and the metastasis site or metastatic status (single vs multiple metastasis) were calculated by using the t-test. A values < 0.05 was considered to be significant. The statistical analyses were performed using the SPSS statistical software package (IBM, Chicago, IL, USA).

## Results

### 3.1 Location of the skeletal metastases

Ninety-three out of the 286 patients (32.5%) with SMUP developed solitary bone metastases, while 193 (67.5%) patients had multiple bone metastases (Table 1A). The ninety-three solitary bone metastases were located in the spine bones (44 cases; 15.4%: cervical spine 3, thoracic spine 13 and lumbar spine 28), extremities (16 cases; 5.6%: femur 4, humerus 4, tibia 8), pelvic bones (16 cases; 5.6%: Iliac 13, pubis 1, ischium 2) and other bones (17 cases; 5.9%: skull 1, rib 6, clavicle 6, scapula 3, lower jaw 1) (Table 1B). In the evaluation of multiple bone metastasis cases, the metastatic sites were similarly divided (spinal, pelvic and extremity) and other areas. In the 193 cases with multiple bone metastases, the multiple bone metastases were restricted to only the spine in 51 (17.8%) cases, only the extremities in 4 (1.4%) cases and only pelvic bones in 3 (1.0%) cases. Multiple bone metastases that occupied the spine and extremities were present in nine (3.1%) cases, the spine and pelvis in 38 (13.3%) cases, the extremities and pelvis in nine (3.1%) cases and all three areas or other areas in 79 (27.6%) cases (Table 1).

**Table 1:** Skeletal metastasis location of SMUP patients.

**A. Metastasis (n = 286)**					
**Location**		***p*** ^***※***^	**Cases**	**%**	**MST(months)**
Solitaly		<0.01	93	32.5%	39.0
Multiple		-	193	67.5%	16.0
**B. Solitaly (n = 93) and Multiple (n = 193)**					
**Location**		**site**	**cases**	**%**	**MST(months)**
Solitaly		Spine	44	15.4%	11.0
		Extremity	16	5.6%	45.0
		Pelvc bone	16	5.6%	43.0
		Other	17	5.9%	37.5
Multiple	Single area	Spine Only	51	17.8%	9.0
		Extremity Only	4	1.4%	39.0
		Pelvis Only	3	1.0%	33.0
	Double area	Spine+Extremity	9	3.1%	12.0
		Spine+Pelvis	38	13.3%	9.5
		Extremity+Pelvis	9	3.1%	14.0
	Triple area and other	Whole Body	79	27.6%	7.0
**C.PS (n = 286)**					
**PS**		***p*** ^***※※***^	**Cases**	**%**	**MST(months)**
0		0.065	12	4.2%	45.0
1		0.065	101	35.3%	27.0
2		0.065	67	23.4%	20.0
3		-	53	18.5%	8.0
4		-	53	18.5%	14.0

*MST*: median survival time

^※^The relationship between solitry metastasis and the multiple metastasis were calculated by using the log-rank test

^※※^The relationship between PS and the metastatic status were calculated by using the t-test.

### 3.2 Performance status (PS)

The performance status of the patients was as follows: PS0, 12 (4.2%); PS1, 101 (35.3%); PS2, 67 (23.4%); PS3, 53 (18.5%) and PS4, 53 (18.7%) (Table 1C). There were no significant differences between the PS and metastasis site, although there were nearly significant differences in the PS and metastatic status. (solitary vs multiple metastases, p = 0.065)

### 3.3 Primary tumors of the SMUP

After performing examinations to identify the primary tumors, the primary sites were detected in 254 of the 286 (88.8%) patients, but were not identified in the remaining 32 (11.2%) patients. With respect to the frequencies of primary tumors, lung cancers were the most frequently detected primary tumors, which were detected in 72 of the 286 patients (25.2%). The other primary tumors were myeloma in 41 (14.3%) patients, prostate cancer in 26 (9.1%), lymphoma in 23 (8.0%), kidney cancer in 18 (6.3%), liver cancer in 12 (4.2%), breast cancer in 12 (4.2%), gastric cancer in 10 (3.5%), pancreatic cancer in 10 (3.5%), thyroid cancer in 9 (3.1%), bile duct/colon cancer in 6 (2.1%), esophageal cancer in 3 (1.0%), thymoma in 2 (0.7%) and there was one case each of submaxillary gland cancer, vaginal cancer, urachal cancer and testicular cancer (0.3% of cases). (Table 2).

**Table 2:** Primary tumors of 286 SMUP patients.

Tumor type	Cases	%	MST(months)	Range(months)
Lung cancer	72	25.2%	9.0	1–58
Myeloma	41	14.3%	105.0	1–131
Unknown	32	11.2%	11.0	1–68
Prostate cancer	26	9.1%	>120	1–128
Lymphoma	23	8.0%	>82	1–82
Kidney cancer	18	6.3%	16.0	1–111
Liver cancer	12	4.2%	7.0	2–14
Breast cancer	12	4.2%	34.0	5–111
Gastric cancer	10	3.5%	12.0	1–59
Pancreatic cancer	10	3.5%	15.0	1–36
Thyroid cancer	9	3.1%	84.0	3–131
Bile duct cancer	6	2.1%	12.0	1–12
Colon cancer	6	2.1%	14.0	2–23
Esophageal cancer	3	1.0%	11.0	3–39
Thymoma	2	0.7%	43.0	10–76
Submaxillary gland cancer	1	0.3%	23.0	23
Vaginal cancer	1	0.3%	31.0	31
Urachal cancer	1	0.3%	12.0	12
Testicular cancer	1	0.3%	120.0	120
Total	286	100.0%	20.0	1–131

*MST*: median survival time

### 3.4 Overall survival

**Fig 1:**
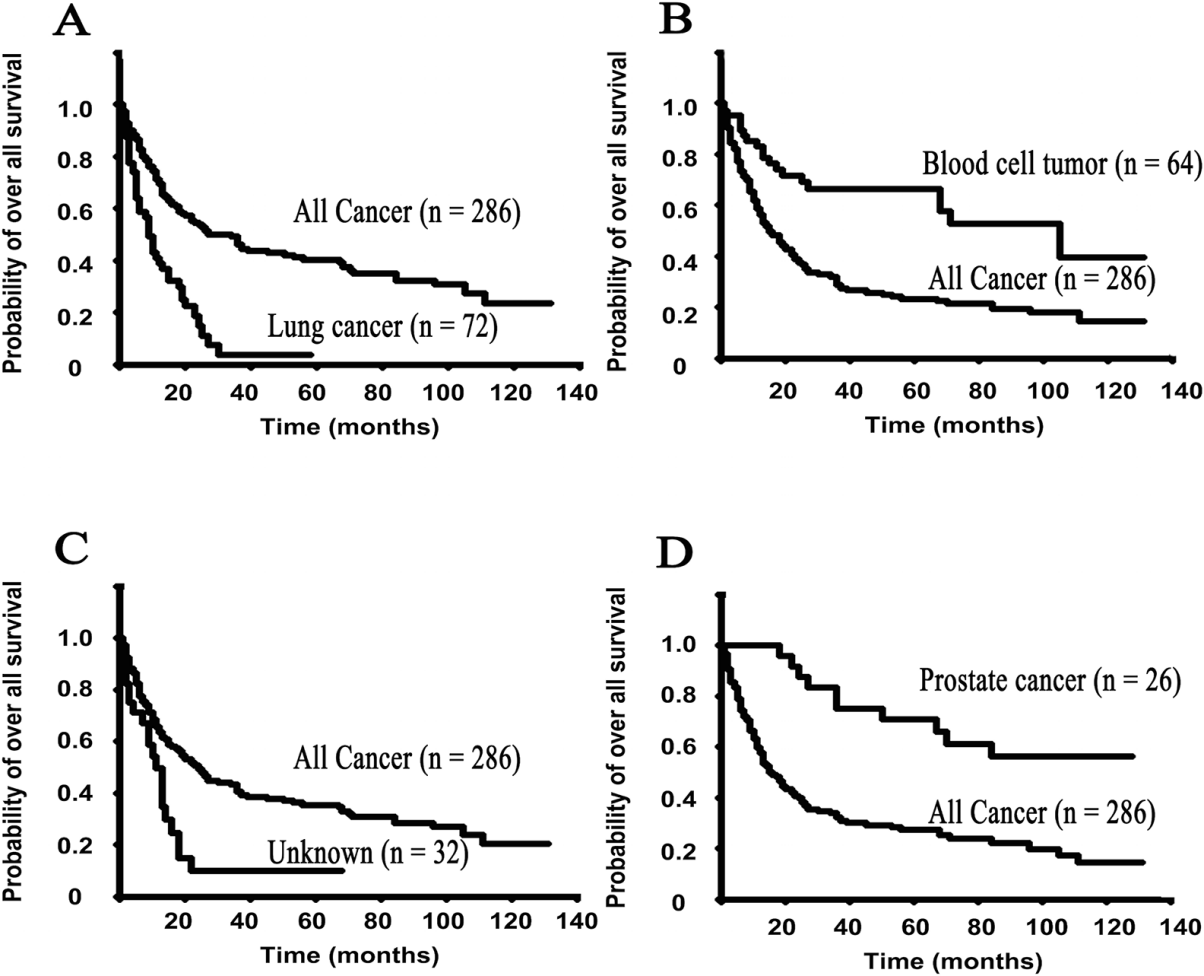
The Kaplan-Meier survival curves show the overall survival rates for each primary site of the SMUP cohort. A: lung cancers, B: blood cell tumors (including multiple myelomas and malignant lymphomas), C: Unknown (still unknown origin after the nine steps) and D: prostate cancers.

The median overall survival was 20.0 months (range, 1 to 131 months) in the 286 SMUP patients. The survival analysis of each primary tumor type revealed that the median survival times were as follows: 9.0 months for lung cancer, 105.0 months for myeloma, 11.0 months for unknown origin, over 120.0 months for prostate cancer, over 82.0 months for lymphoma, 16.0 months for kidney cancer, 7.0 months for liver cancer, 34.0 months for breast cancer, 12.0 months for gastric cancer, 15.0 months for pancreatic cancer, 84.0 months for thyroid cancer, 12.0 months for bile duct cancer, 14.0 months for colon cancer, 11.0 months for esophageal cancer, 43.0 months for thymoma, 23.0 months for submaxillary gland cancer, 31.0 months for vaginal cancer, 12 months for urachal cancer and 120.0 months for testicular cancer (Fig 1A–1D, Table 2).

**Fig 2:**
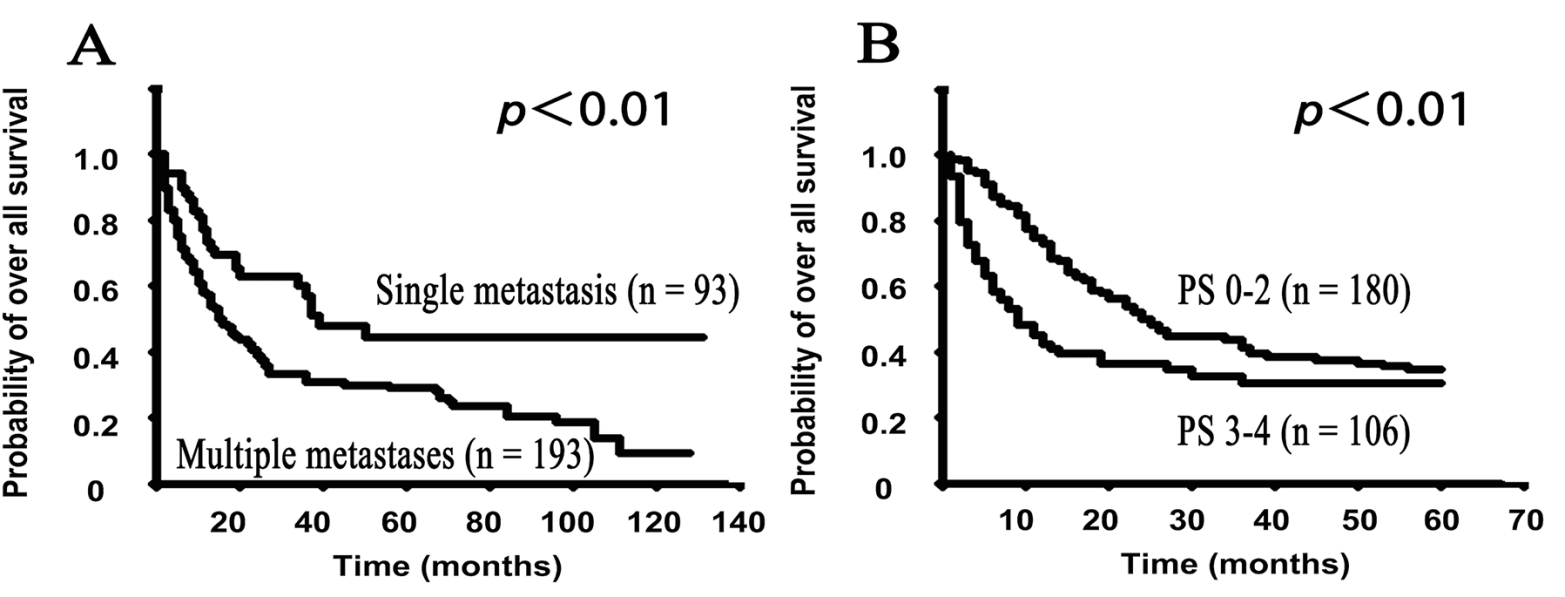
The Kaplan-Meier survival curves show the survival rates regarding skeletal metastases and the performance status (PS) at the first visit in the SMUP cohort. A: The different skeletal metastasis situations (solitary metastases vs. multiple metastases), B: PS (good PS of 0–2 vs. poor PS of 3–4). A, The Kaplan-Meier curve of the overall survival demonstrated that patients with solitary metastases (MST: 16.0 months) had a longer survival than those with multiple metastases (MST: 39.0 months) (p<0.01). B. To elucidate the early survival rates based on the PS, we employed the limited survival data within five years of follow-up in all 286 SMUP cases, and the data were analyzed using the Kaplan-Meier method. The limited data showed that the good PS group had a better prognosis compared to the poor PS group, and there was a significant difference in the survival of the good and poor PS groups (p<0.01). The non-limited survival data with regard to the PS are shown in S2 Fig

Furthermore, the median overall survival time in the patients with solitary bone metastasis was 39.0 months, and that in patients with multiple bone metastases was 16.0 months. There was a statistically significant difference in the median overall survival time between these two groups (Table 1A and Fig 2A, p<0.01). The median survival times for the other situations regarding bone metastases are shown in Table 1B.

The overall survival rates according to the performance status (PS) were also analyzed. The median overall survival of patients with a PS of 0 was 45.0 months, while the rates were 27.0 months for PS1, 20.0 months for PS2, 8.0 months for PS3 and 14.0 months for PS4 (Table 1C). To evaluate the associations between the PS and survival, we classified these PS grades into two groups (Good PS: 0–2 and Poor PS: 3–4) and then analyzed the survival. There was a statistically significant difference in the overall survival rate between these two groups within first five years (Fig 2B, p<0.01), however, this difference was lost after five years (S1 Fig).

### 3.5 Diagnostic abilities of each step for identifying primary tumors

**Fig 3:**
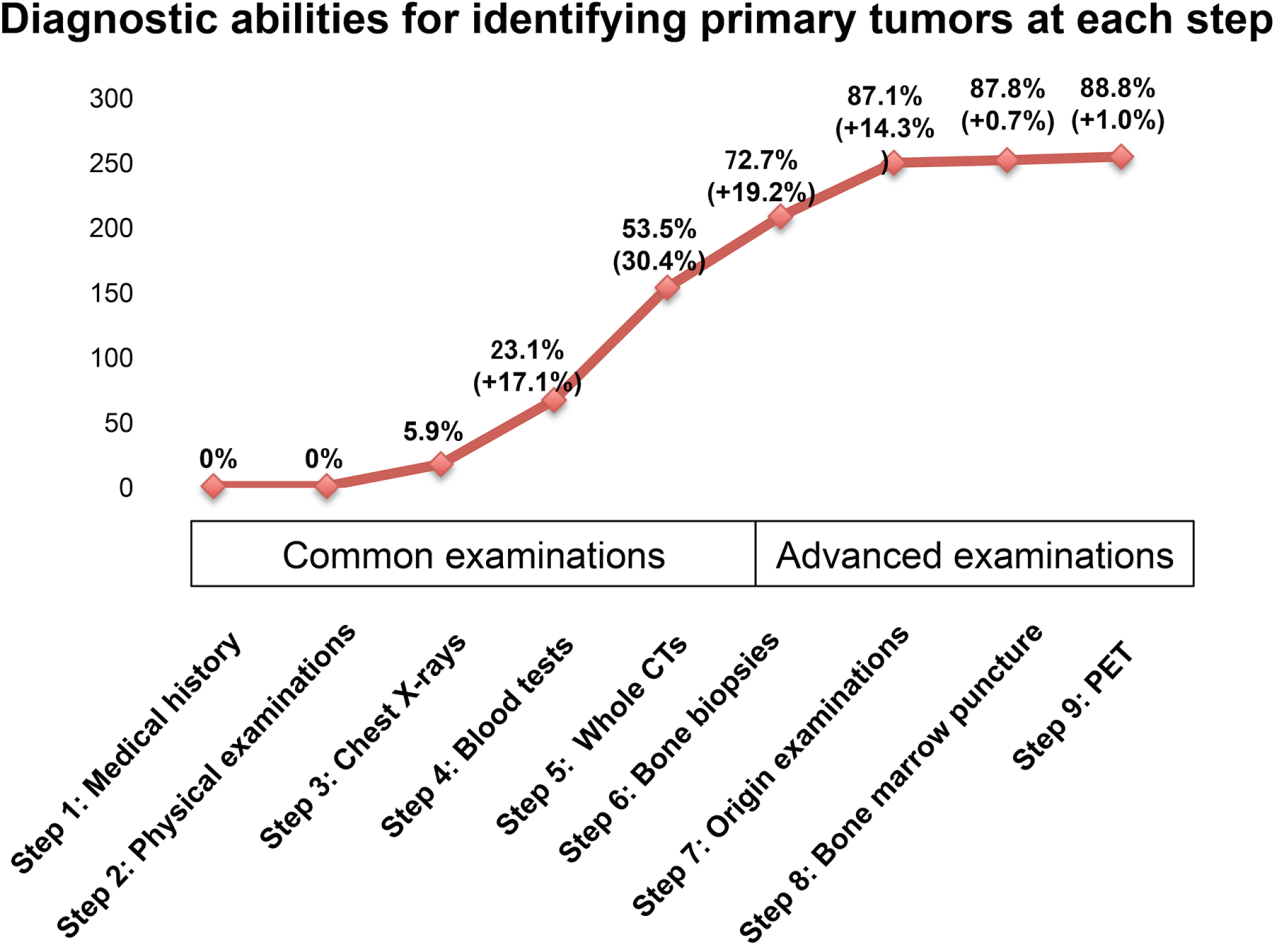
The diagnostic abilities of each step for identifying the primary tumors in 286 SMUP cases: The graph shows the diagnostic abilities of each examination step. We divided the steps into two categories: steps 1~5 as “common examinations”, and steps 6~9 as “advanced examinations”. Almost half of the SMUP cases (53.3%) were diagnosed by “common examinations”. Additionally, Step 9 (PET scan) was not efficient for identifying the primary site in our cohort. The details of these findings are shown in S2 Fig and Supplemental Table A in S1 File.

Next, we investigated the diagnostic abilities of each step (Fig 3, S2 Fig) for identifying the primary tumor. Step 1: only medical history-taking did not identify the primary site in any of the patients (0/286; 0%). In Step 2, physical examinations were also unable to identify the primary site in any of the patients (0/286, 0%). In Step 3, the chest X-rays identified the primary tumor in 17/286 (5.9%) cases. In Step 4, the blood tests identified the primary tumors in 49/286 (17.1%) cases, while in Step 5, the whole CT scans identified the primary tumors in 87/286 (30.4%) cases. In Step 6, the first of the advanced examinations, bone biopsies identified the primary tumor in 55/286 (19.2%) cases. In Step 7, origin examinations and origin biopsies identified the primary tumors in 41/286 (14.3%) cases. In the last two steps, the BMP in Step 8 identified the primary tumor in 2/286 (0.7%) cases, and the PET scans in Step 9 identified the primary tumors in 3/286 (1.0%) cases (Fig 3, S2 Fig). The details of the contents of these findings are shown in Supplemental Table A in S1 File.

### 3.6 The rates of positive findings for each examination

We also investigated the detection rates of each examination retrospectively.

#### a. Medical history

In the medical history-taking step, we obtained seven positive findings in 286 patients. All seven had prior histories of cancers, including two cases of gastric cancer, two of thyroid cancer, two of breast cancer and one of testicular cancer, all of which had occurred over 15 years earlier (Supplemental Table B1 in S1 File). However, we could not define the origin sites in this step.

#### b. Physical examination

In the physical examinations, we had five cases with positive findings, including four breast cancers and one thyroid cancer in the 286 patients (S2 Fig). However, we could not define the origin sites in this step.

#### c. Chest X-ray

Chest plain radiographs were obtained for all 286 patients. These studies identified 34 (11.9%) positive findings of primary tumors, which consisted of 33 lung cancers and one thymoma. 33 (45.8%) of the total of 72 lung cancers that were the primary tumors had positive findings in the chest X-rays (Table 3A).

**Table 3:** Positive findings of clinical examination.

**A. Positive findings regarding primary site of X-ray in 286 cases (34 positive findings)**				
	Primary site	Positive cases	Total number of examination in each tumor type	Detection rate
Chest X-ray	Lung	33	Lung cancer (72)	45.8%
	Thymic gland	1	Thymoma (2)	50.0%
**B. Positive findings of tumor markers in 286 cases (133 positive findings)**				
Tumor marker	Positive cases	Positive rate	Tumor type	
CEA	25	8.7%	Lung cancer 14, Colon cancer 3, Pancreatic cancer 3, Gastric cancer 2, Breast cancer 2, Prostate cancer 1	
PSA	24	8.4%	Prostate cancer24	
CA19-9	22	7.7%	Pancreatic cancer 7, Gastric cancer 6, Lung cancer 4, Colon cancer3, Bile duct cancer1, Kidney cancer1	
AFP	7	2.4%	Liver cancer 6, Bile duct cancer 1	
PIVKA-Ⅱ	6	2.1%	Liver cancer 6	
IL-2	19	6.6%	Lymphoma 9,Unknown cancer 2, Lung cancer 2, Myeloma 2, Kidney cancer 1, Esophageal cancer 1, Liver cancer 1, Gastric cancer 1	
IEP	30	10.5%	Myeloma 30	
**C. Positive findings regarding primary site of whole CT in 282 cases (149 cases)**				
				
	Primary site	Povitive cases	Total number of examination in each tumor type	Detection rate
Chest CT	Lung	64	Lung cancer(72)	88.9%
	Breast	11	Breast cancer (12)	91.7%
	Thyroid	5	Thyroid cancer (9)	55.6%
	Thymic gland	2	Thymoma (2)	100.0%
	Esophagus	2	Esophageal cancer(3)	66.7%
	Submaximally Gland	1	Submaximally Gland cancer(2)	100.0%
Abdomen-pelvic CT	Kidney	17	Kidney cancer(18)	94.4%
	Prostate	13	Prostate cancer(23)	56.5%
	Liver	12	Liver cancer(12)	100.0%
	Pancreas	6	Pancreatic cancer(10)	60.0%
	Stomach	6	Gastric cancer(10)	60.0%
	Bile Duct	6	Bile Duct cancer(6)	100.0%
	Colon	4	Colon cancer(6)	66.7%

#### d. Tumor markers

The values for PSA were examined for male patients, and examinations of other tumor markers were performed for all 286 patients. Our previous study evaluated the sensitivities of tumor markers, including CEA, PSA and AFP, and demonstrated that the tumor markers were abnormally elevated in 73% of SMUP patients [1]. Based on the previous study, we adopted the tumor markers which were more than 100 units as an abnormal value to identify the primary tumors. We also adopted a value for IL-2 of 1000 units as an abnormal value, and we use the standard value of IEP. These examinations identified 133 positive findings in 286 patients.

The CEA level was abnormally elevated in 25 (8.7%) cases, leading to the diagnosis of 14 lung cancers, 3 pancreatic/colon cancers, 2 gastric/breast cancers and 1 prostate cancer. The level of PSA was abnormally elevated in 24 (8.4%) cases (all of which had prostate cancer), CA19-9 was elevated in 22 (7.7%) cases (7 pancreatic cancers, 6 gastric cancers, 4 lung cancers, 3 colon cancers, 1 bile duct/kidney cancer), AFP was elevated in 7 (2.4%) cases (6 liver cancers and 1 bile duct cancer), PIVKA-II was elevated in 6 (2.1%) cases (6 liver cancers), IL-2 was elevated in 19 (6.6%) cases (9 lymphomas, 2 cases each with unknown tumors, lung cancers and myeloma, and 1 case each of kidney, esophageal, liver and gastric cancer) and IEP was elevated in 30 (10.5%) cases (all of which had myelomas) (Table 3B).

With regard to the sensitivity of detecting each tumor, 24 of 26 (92.3%) prostate cancers had positive findings for PSA, 30 of 41 (73.2%) multiple myelomas had positive findings for IEP, 6 of 12 (50.0%) liver cancers had positive findings for AFP, 6 of 12 (50.0%) liver cancers had positive findings for PIVKA-II and 9 of 23 (39.1%) lymphomas had positive findings for IL-2 (Supplemental Table C in S1 File).

#### e. CT scans

Whole CT scans were performed for 282 of 286 cases, excluding three cases with prostate cancers rapidly diagnosed by PSA and one case of lung cancer diagnosed by chest X-ray who changed hospitals before the CT examination. These imaging studies identified 149 positive findings of primary tumors and 109 positive findings other than the primary lesion.

The chest CTs identified lung cancer (64 cases), breast cancer (11 cases), thyroid cancer (9 cases), thymoma (2 cases), esophagus cancer (2 cases) and submaxillary gland cancer (1 case). The abdomen-pelvic CTs identified kidney cancer (17 cases), prostate cancer (13 cases), liver cancer (12 cases), pancreatic cancer (6 cases), gastric cancer (6 cases), bile duct cancer (6 cases) and colon cancer (4 cases)(Table 3C and Supplemental Table D in S1 File).

With regard to each type of tumor, 64 of 72 (88.9%) lung cancers, 11 of 12 (91.7%) breast cancers, 5 of 9 (55.6%) thyroid cancers, 2 of 2 (50.0%) thymomas, 2 of 3 esophageal cancers and 1 of 1 (100.0%) submaxillary gland cancers had positive findings in the chest CTs. Furthermore, 17 of 18 (94.4%) kidney cancers, 13 of 23 (56.5%) prostate cancers, 12 of 12 (100.0%) liver cancers, 6 of 10 (60.0%) gastric/pancreatic cancers, 6 of 6 (100%) bile-duct cancers and 4 of 6 colon cancers had positive findings in the abdominal-pelvic CTs (Table 3C).

The details of the positive findings of non-primary lesions are shown in Supplemental Table D in S1 File.

#### f. Bone biopsies

Bone biopsies from the metastatic site were performed in 141 patients. These examinations had positive findings in 138 of 141 cases (97.9%). These histological diagnoses consisted of adenocarcinoma (42 cases), myeloma (26 cases), undifferentiated carcinoma (19 cases), B-cell lymphoma (12 cases), squamous cell carcinoma (8 cases), follicular lymphoma (7 cases), clear cell carcinoma (6 cases), neuroendocrine carcinoma (4 cases), hepatocellular cancer (3 cases), Hodgkin’s lymphoma (2 cases), large cell carcinoma (2 cases), Ki-1 lymphoma (1 case), transitional cell carcinoma (1 case), papillary carcinoma (1 case), germ cell tumor (1 case), adenosquamous carcinoma (1 case), small cell carcinoma (1 case) and carcinoma (1 case) (Table 4). The details of the primary sites are shown in Table 4.

**Table 4:** Histrological diagnoses of bone biopsy in 141 cases.

Histrogical type	Positive cases	Tumor type
Adenocarcinoma	42	Unknown 12, Lung cancer 9, Prostate cancer 7, Pancreatic cancer 3, Gastric cancer 2, Colon cancer 2, Liver cancer 2, Bill duct cancer 1, Kidney cancer 1, Vaginal cancer 1, Esophageal cancer 1, Urachal cancer 1
Myeloma	26	Myeloma 26
Undifferentiated carcinoma	19	Lung cancer 8, Unknown 7, Kidney cancer 3, Thymoma 1
B-cell lymphoma	12	Lymphoma 12
Squamous cell carcinoma	8	Lung cancer 3, Unknown 3, Submaxillary gland cancer 1, Esophageal cancer 1
Follicular carcinoma	7	Thyroid cancer 6, Lymphoma 1
Clear cell carcinoma	6	Kidney cancer 5, Liver cancer 1
Neuroendocrine carcinoma	4	Unknown 1, Breast cancer 1, Lung cancer 1, Thymoma 1
Hepatocellular carcinoma	3	Liver cancer 3
Hodgikin Lymphoma	2	Lymphoma 2
Large cell carcinoma	2	Lung cancer 2
Ki-1 Lymphoma	1	Lymphoma 1
Transitional cell carcinoma	1	Kidney cancer 1
Papillary carcinoma	1	Thyroid cancer 1
Germ cell carcinoma	1	Testicular cancer 1
Adenosquamous carcinoma	1	Breast cancer 1
Small cell carcinoma	1	Lung cancer 1
carcinoma	1	Breast cancer 1
Sampling error	3	Lymphoma 1, Unknown 1, Kidney cancer 1
Total	141	

#### g. Examinations for suspected sites of origin

The examinations performed for suspected sites of origin included specific imaging studies, such as thyroid gland echo, mammography and gastric/colon fiberscopy, followed by biopsies when necessary.

With regard to these specific imaging studies for each primary site, gastric fiberscopy, colon fiberscopy, mammography and thyroid echo studies were performed in 60 cases, 33 cases, 18 cases and 20 cases, respectively. Gastric fiberscopy was performed in nine patients with suspected gastric cancer and three with suspected esophageal cancer, and the tumors of origin were identified in all 12 (100%) cases. Colon fiberscopy was carried out in six suspected colon cancer patients, and tumors were detected all six (100%) patients. Mammography was performed in six suspected breast cancer patients, and identified the tumors in all six (100%) patients. Thyroid echo were carried out in nine suspected thyroid cancer patients, and positive findings were detected in seven (77.8%) of these patients (Table 5A).

**Table 5:** Special examinations.

**A Special examinations of image studies**			
**Examination**	**Positive cases**	**Total number of examination in each tumor type**	**Detection rate (for target)**
Gastric fiber	Stomach (9) Esophagus (3)	Gastric cancer (9) Esophageal cancer(3)	100%
Colon fiber	Colon (6)	Colon cancer(6)	100%
Mammography	Breast (6)	Breast cancer(6)	100%
Thyroid echo	Thyroid (7)	Thyroid cancer (9)	77.8%
**B Origin biopsy in 122 cases (110 case had positive findings)**			
**Biopsy site**	**positive cases**	**Total number of examination in each tumor type**	**Detection rate**
Lung	33	Lung cancer 37	89.2%
Prostate	23	Prostate cancer 23	100.0%
Lymph node	12	Lymphoma 13	92.3%
Breast	9	Braast cancer 9	100.0%
Gastro	9	Gastric cancer 9	100.0%
Colon	5	Colon cancer 5	100.0%
Kidney	4	Kidney cancer 5	80.0%
Thyroid	4	Throid cancer 9	44.4%
Liver	3	Liver cancer 3	100.0%
Esophagus	3	Esophageal cancerc3	100.0%
Bile Duct	2	Bile duct cancer 2	100.0%
Thymus Gland	1	Thymoma 1	100.0%
Urachus1	1	Urachal cancer 1	100.0%
Submaximally Gland	1	Submaximally gland cancer 1	100.0%
Pancreas	0	Pancreatic cancer 1	0.0%

Biopsy samples were obtained from 122 patients. These examinations had positive findings in 110/122 (90.2%) patients as follows: lung cancer 33/37 (89.2%), prostate cancer 23/23 (100%), lymphoma 12/13 (92.3%), breast cancer 9/9 (100%), gastric cancer 9/9 (100%), colon cancer 5/5 (100%), kidney cancer 4/5 (80.0%), liver cancer 3/3 (10.0%), thyroid cancer 4/9 (44.4%), esophageal cancer 3/3(100%), bile duct cancer 2/2 (100%), thymoma 1/1 (100%), urachal cancer 1/1(100%), submaxillary gland cancer 1/1 (100%) and pancreatic cancer 0/1(0%) (Table 5B).

#### h. BMP

BMP was performed in 42 patients. These studies had positive findings in 24/42 (57.1%) cases, with 21 myelomas and three lymphomas detected. Furthermore, BMP resulted in the final diagnosis in 21 (77.8%) of 27 myeloma patients, and in 3 (37.5%) of the 8 lymphoma patients (Supplemental Table E in S1 File).

#### i. PET scans

PET scans were performed in 80 patients, and positive findings were obtained in 30/80 (37.5%) patients as follows: lung cancer 18/20 (90.0%) cases, gastric cancer 2/5 (40.0%), lymphoma 1/7 (14.3%), kidney cancer 1/4 (25.0%), colon cancer 1/3 (33.3%), thyroid cancer ½ (50.0%), liver cancer 1/1 (100%), thymoma 1/1(100%), submaxillary gland cancer 1/1 (100%), urachal cancer 1/1 (100%), breast cancer 1/1 (100%) and vaginal cancer 1/1 (100%). On the other hand, the PET scans indicated negative findings to rule out the presence of a tumor as follows: unknown 0/18 (0%), myeloma 0/8 (0%), prostate cancer 0/2 (0%), breast cancer 0/2 (0%), pancreatic cancer 0/2 (0%), testicular cancer 0/1 (0%) (Table 6).

**Table 6:** PET in 80 cases.

	Tumor type of positive	Positive cases	Total number of examination in each tumor type	Detection rate
**PET (positive)**	Lung cancer	18	20	90.0%
	Gastric cancer	2	5	40.0%
	Lymphoma	1	7	14.3%
	Kidney cancer	1	4	25.0%
	Colon cancer	1	3	33.3%
	Thyroid cancer	1	2	50.0%
	Liver cancer	1	1	100.0%
	Thymoma	1	1	100.0%
	Submaxially gland cancer	1	1	100.0%
	Urachal cancer	1	1	100.0%
	Breast cancer	1	1	100.0%
	Vaginal cancer	1	1	100.0%
**PET (negative)**	Unknown	0	18	0.0%
	Myeloma	0	8	0.0%
	Prostate cancer	0	2	0.0%
	Breast cancer	0	2	0.0%
	Pancreatic cancer	0	2	0.0%
	Testicular cancer	0	1	0.0%

## Discussion

From 2002 to 2014, we treated 2,641 patients with bone metastasis, and among them, we found 286 patients (10.8%) in which the primary sites remained unknown at the first visit. This frequency is lower to more than in the previous studies [1, 4, 5]. In this study, we were able to identify 254 primary lesions (88.8%) in these 286 SMUP patients. This detection rate was equal to that in our previous report [1]. However, there were big differences in the detection rates between our study and older studies of patients treated between 1970 and 1995 [6–12]. With respect to the most common primary tumors, our data indicated that lung cancer (72 patients; 25.2%) was the most frequently identified primary tumor. Blood cell tumors, including multiple myeloma (41 patients; 14.3%) and lymphomas (23 patients; 8.0%), as well as prostate cancer (26 patients; 9.1%) were also common in our study. These findings are consistent with the previous reports suggesting that lung cancers and prostate cancers were the most common primary tumors [1, 4, 8, 9]. The previous studies often excluded blood cell tumors, although our present study included them, since we wanted to clarify the exact rates of the various tumors which cause abnormal bone lesions encountered during the patients’ first visit. Gynecological carcinomas are common, although in our pervious paper, there were no gynecological carcinomas identified as the primary lesions [1]. Therefore, examinations of the cervix and uterus were not conducted as a routine part of the evaluation. Some of the SMUP cases finally categorized as unknown origin in this study might have been derived from gynecological cancers.

It is notable that our data are the first to provide the estimated survival time of SMUP patients for each tumor type. Previous studies have not shown any data regarding the patients’ survival. This is critical information for clinicians to determine whether the patients should be subjected to aggressive treatments with surgery. In this study, we found that the median survival of the patients whose primary remained unknown at the final diagnosis was about 11.0 months, which was similar to the rates of patients with lung cancer and gastric cancer. These results indicate that the patients with unknown primary tumors should be treated promptly with palliative treatment, including zoledronic acid, radiation and pain control, to maintain their performance status and to avoid skeletal related events. Lengthy evaluations to determine the primary site prior to treatment should be avoided.

Regarding the location of bone metastases, we found that the median survival time in patients with solitary bone metastasis (39 months) was significantly longer than that of the patients with multiple bone metastases (16.0 months). In addition, we also clarified that the patients with single bone metastases located on the extremities or pelvis had a longer survival (median: over 43 months) than those with metastases in the spine (11 months). These findings suggest that SMUP patients with a single bone metastasis in the extremities or pelvis may respond well to surgical treatments. Furthermore, SMUP patients proven to have prostate cancers, blood cell tumors, thyroid cancers and breast cancers show longer survival times compared to those with other cancers. Therefore, clinicians may be able to treat these SMUP patients with intensive surgeries if there are adaptive operations. With respect to the survival and PS, there were no significant differences in the overall survival between the patients with a good PS and those with a poor PS. However, within the first five years of the diagnosis, patients with a good PS had a significantly longer survival than those with a poor PS (Fig 2B). In our previous study that focused on prognosis factors in skeletal metastasis patients using the whole cohort (not only SMUP), the analyses also indicated that prostate cancers, blood cell tumors, thyroid cancers, breast cancers and good PS were favorable prognosis factors in comparing to the other clinical situations [13]. These results indicate that treatments should be started to maintain the PS in SMUP patients, even before the detection of the primary sites.

Chest and abdominal-pelvis CT scans were the most useful modalities to identify the primary tumor sites. Whole CT scans identified 149 (52.1%) primary sites of the 286 SMUP cases. Chest CT detected 64 (88.9%) of 72 lung cancers and 11 (91.7%) of 12 breast cancers. These results indicate that chest CT was the most essential imaging study for SMUP derived from undiscovered tumors in this area. Abdominal-pelvic CT scans found 17 (94.4%) of 18 kidney cancers, all 12 (100%) liver cancers and all 6 (100%) bile-duct cancers, so the sensitivity of detection of CT for these tumors was excellent. These results indicate that CT scans should be performed to identify liver and kidney cancers. However, regarding prostate cancers, CT scanning detected only 13 (56.5%) of 23 prostate cancers. Compared to the tumor marker PSA, which detected 24 (92.3%) of 26 prostate cancers, these CT scans were not sensitive. Therefore, for male SMUP patients, it is recommended that the levels of PSA be checked first, and that CT scans should be used to confirm the PSA findings.

We described that it is not appropriate to start with a skeletal biopsy for SMUP patients in our previous report, because the detection rates of the primary sites were low. However, our previous report did not include patients with multiple myeloma or lymphoma [1]. In this study, our cohort included cases of both multiple myeloma and lymphoma, so we analyzed anew the data obtained from bone biopsies. Naturally, evaluating the levels of IEP and IL-2 was the most efficient way to identify the multiple myelomas and lymphomas. However, these two tumor markers could not completely cover all SMUP patients who had blood cell tumors as screening tests. Therefore, bone biopsies may help to detect these tumors. In some tumors, such as thyroid cancers and myelogenic malignancies, the specific histological features help to identify the primary site, therefore, bone biopsies can be crucial to identify the primary sites in patients with these cancers. Blood cell tumors have not usually been categorized to cause skeletal metastases, because these tumors originally develop in bones. Therefore, our previous study that excluded these blood cell tumors had a more precise definition of "skeletal metastases" than this study. However, the aim of this study was to establish a prompt and definite diagnosis and treatment strategies for patients who had bone irregularities and were suspected to have skeletal metastasis in the clinical setting. Such patients usually include those with blood cell tumors. Therefore, we included these tumors and analyzed them together in this study.

Our previous study also described that routine laboratory analyses were important for the general evaluation of the patient’s condition, but did not contribute to identifying the primary lesion [1]. In addition, that study reported that the tumor marker levels were abnormally elevated in 73% of patients with solid carcinomas or adenocarcinomas of unknown primary site [1]. Therefore, we adopted a value of tumor markers over 100 units as abnormal values to identify the primary tumors, and we omitted the results of routine laboratory analyses, including the blood cell count, blood chemistry findings and urinalysis in this study. Our results showed that high values of PSA were associated with prostate cancers (92.3%), and that positive cases of IEP were associated with multiple myeloma (73.2%). Furthermore, AFP and PIVKA-II also had specific relationships to liver cancers (50.0% and 50.0%, respectively) (Supplemental Table C in S1 File). PSA had the highest specificity as a tumor marker in this study. The prostate cancer mortality was reduced after the introduction of PSA screening [14–16]. In addition, PSA plays an important role in the spread and malignancy of prostate cancer [17], and in the judgment of the effects of treatment and disease recurrence [18]. However, because mild elevation of the PSA level is seen even in patients with benign prostatic hyperplasia, it has a low detection rate. These issues need to be kept in mind when diagnosing prostatic cancers using the value of PSA.

In the myeloma cases, the IEP level was a useful marker. We also retrospectively reviewed the findings of the Bence-Jones protein in the urinalysis. A urinalysis was performed in 24 myeloma patients, and we found that 14 (58.3%) of the 24 patients had positive findings. Furthermore, we also found four positive cases that were finally diagnosed as not being multiple myelomas (lymphoma, liver cancer, prostate cancer and thyroid cancer). B-J protein is generally detectable in patients with multiple myeloma, malignant lymphoma and amyloidosis [19]. There are no detailed data available about solid tumors in the previous reports. Based on our present results, some solid cancers also had positive values. Therefore, this issue needs to be considered when the Bence-Jones protein test is used.

Regarding IL-2, this tumor marker identified 9 (39.1%) of 23 lymphomas (Supplemental table C in S1 File) and detected ten SMUP patients who did not have lymphomas as false positives (Table 3B). The primary tumors in above ten SMUP patients with elevated of IL-2 levels were lung cancer, myeloma, kidney cancer, esophageal cancer, liver cancer or gastric cancer. Increased concentrations of serum soluble IL-2 have been found in patients with diverse diseases, including lymphoma, leukemia, sarcoidosis, tuberculosis, viral infection, autoimmune diseases, and organ allograft rejection [20–22]. However, elevated IL-2 levels have previously been reported in cases of solid tumors of lymph node metastasis, including gastric cancer and lung cancer [23, 24]. Based on our findings, IL-2 appears to be a crucial tumor marker to detect lymphoma, but the test also showed a high rate of false positive findings. Therefore, origin testing and bone biopsies may be necessary to confirm whether positive cases truly have lymphoma.

With respect to gastric and colon fiberscopy, these examinations detected all nine gastric cancers, all three esophageal cancer and all six colon cancers. On the other hand, six of ten gastric cancers (60.0%), two of three esophageal cancers (66.7%) and four of six colon cancers (66.7%) could be identified by CT scans. Therefore, these examinations should not be performed as the first-line (common examination) imaging studies, because the frequency of colon cancers in SMUP was only 2.1% (6/286 SMUPs) and these examinations are stressful tests. Mammography identified all six breast cancers in the patients evaluated, and was thought to be useful to detect the breast cancers. The chest CT studies could similarly identify all nine breast cancers evaluated. Therefore, mammography may have supportive role to confirm the presence of the lesions.

Regarding abdominal lesions, our previous study described that CT is useful for detecting abdominal lesions, although the abdominal echo examination was also effective and noninvasive. The present study also indicated that abdominal CT could detect all 12 liver cancers. We think that abdominal echo could be used during the origin biopsy, and might provide additional information. With respect to ultrasound examination of the thyroid, five (55.6%) of nine thyroid cancers were detected by neck CT, and seven (77.8%) of nine thyroid cancers were detected by ultrasound. These results suggest that it is necessary to perform both whole CTs and ultrasound of the neck when a patient is suspected to have thyroid cancer.

In this study, PET scanning was used as a final tool to identify the primary lesion, since it is more expensive and not available in most general hospitals. Therefore, in this series, we performed PET scans only in limited patients, even if the primary sites were not identified by Step 8. In our study, we performed PET scans for 21 SMUP patients with unknown primary sites in step 9, and the results showed PET scanning could identify the primary site in only 3 of the 21 cases. Based on our data, PET scanning may be not be efficient to identify the primary sites. Other studies have similarly concluded that CT scans may be more useful than PET scans for determining the primary lesions of a bone metastasis [25].

## Conclusions

We performed retrospective analyses including 286 SMUP patients to establish the optimal diagnostic strategy and to elucidate the survival rates of these patients. We found that our examinations could detect 254 (88.8%) primary sites, and the “common examinations” (up to the fifth step) could detect approximately half of the primary site in our SMUP cohort. We also found that the overall survival rate for the overall SMUP patients was 11.0 months. The survival rate of the patients with an unknown primary (still undiagnosed after the nine steps, 32 patients) was close to the survival time of SMUP groups with poor prognosis finally diagnosed as primary lung cancers and gastric cancers. We believe that the findings of our study would contribute to establishing optimal strategies for diagnosing the primary site in SMUP patients, and our data also provide definite estimates of the survival times of each SMUP situation. We believe that these diagnostic strategies and the survival rates may help clinicians to provide better treatments to the SMUP patients.

Each survival curve was compared with that of the total 286 SMUP cases. A: Seventy-two of the 286 SMUP cases were diagnosed to have lung cancers, and the median survival time (MST) was 9.0 months. B: Sixty-four of the 286 SMUP cases were defined as having blood cell tumors, and the MST was 105 months (41 multiple myelomas: MST, 105 months and 23 malignant lymphomas: MST, over 82 months). C: Thirty-two of the 286 SMUP cases still did not have a known primary site, and these cases were defined as having an “unknown primary”. The MST of the “unknown primary” group was 11.0 months. D: Twenty-six of the 286 SMUP cases were diagnosed to have prostate cancers, and the MST was over 120 months.

In our cohort, the prostate and blood cell tumors were associated with longer overall survival rates in comparison to the other cancers. On the other hand, lung cancers (MST: 9.0 months), liver cancers (MST: 7.0 months), gastric cancers (MST: 12.0 months), bile-ductal cancers (MST: 12.0 months) and esophageal cancers (MST: 11.0 months) were considered to be part of the poor prognosis group, and the MST of the “unknown primary” cases was similar to those of this poor prognosis groups.

## Supporting Information

S1 Fig The Kaplan-Meier survival curves show the overall survival based on the PS at first visit in the SMUP cohort. There were no significant differences in the survival between the good PS group and the poor PS group in the overall cohort.
(TIF)

S2 Fig Flow charts of our diagnostic steps for identifying primary tumors. (TIF)

S1 File Supplemental Table A. The detection rates in each step for determining final diagnosis of primary sites. Supplemental Table B1, B2. Medical histories and physical examinations in 286 cases. Supplemental Table C. Positive findings of tumor specific markers in 227 cases. Supplemental Table D. Positive findings without non-primary lesions of whole CT in 286 cases. Supplemental Table E. Bone Marrow Puncture in 42 patients. Supplemental Table F. Patients characteristics.
(XLSX)
