# Supplementary figures and images for: Skeletal Metastasis of Unknown Primary Origin at the Initial Visit: A Retrospective Analysis of 286 Cases
Source: PLoS One. 2015 Jun 26;10(6):e0129428. doi: 10.1371/journal.pone.0129428 (PMC4482691; doi:10.1371/journal.pone.0129428)

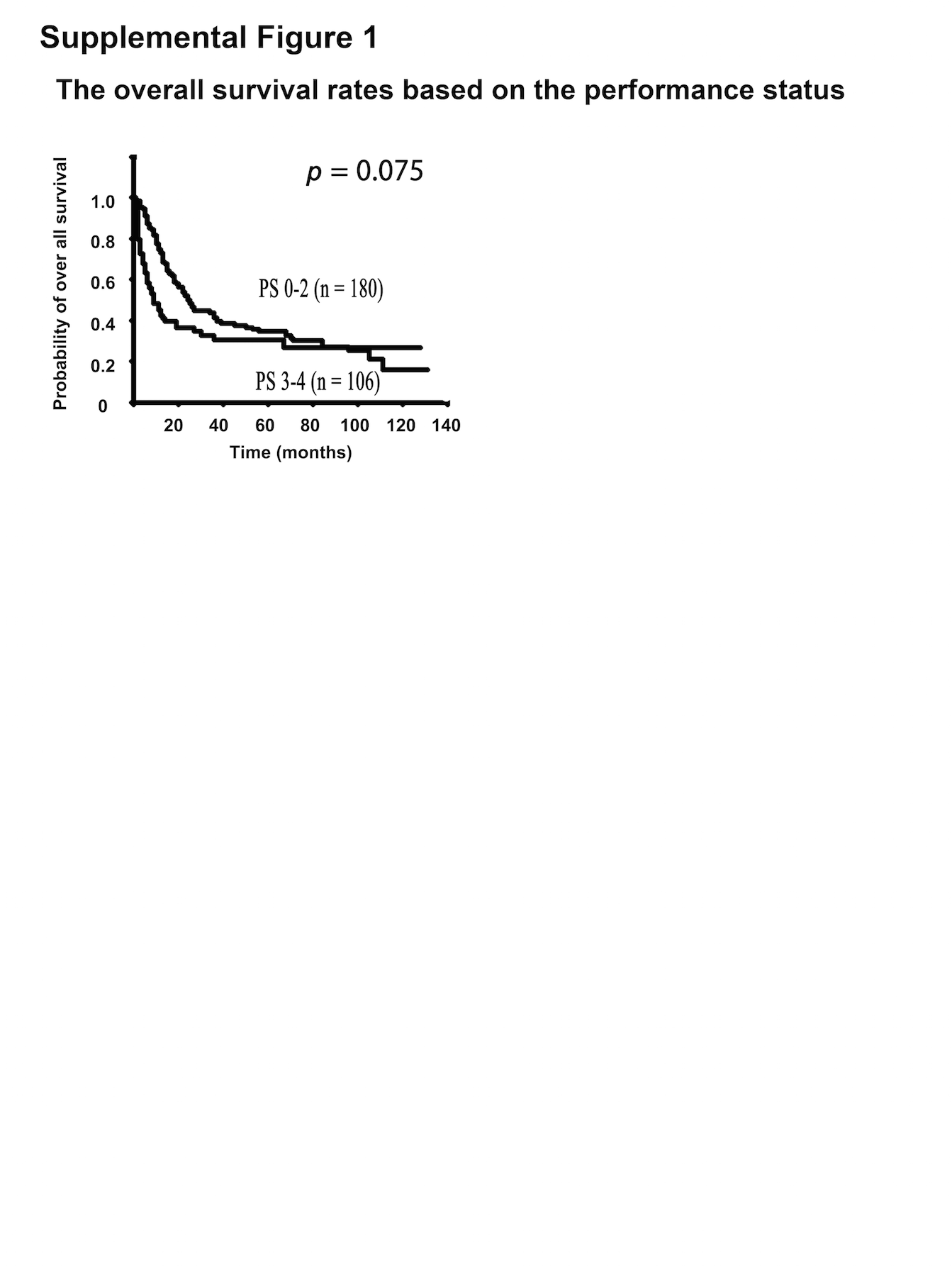

Supplement: S1 Fig — There were no significant differences in the survival between the good PS group and the poor PS group in the overall cohort. (TIF) [file pone.0129428.s001.tif]

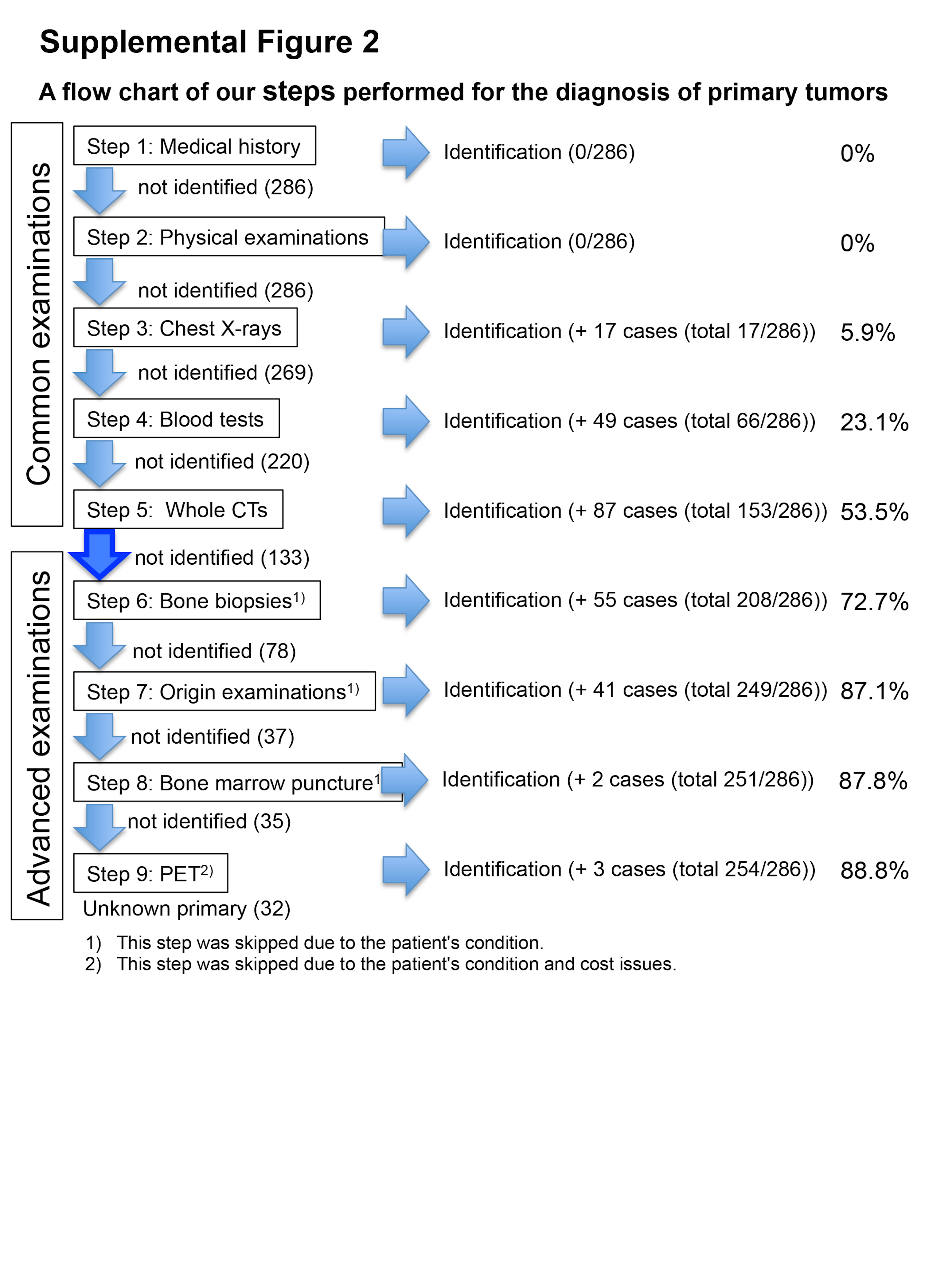

Supplement: S2 Fig — (TIF) [file pone.0129428.s002.tif]
